# Hantavirus Infection in Anajatuba, Maranhão, Brazil

**DOI:** 10.3201/eid1008.040002

**Published:** 2004-08

**Authors:** Wellington S. Mendes, Antônio A.M. da Silva, Luís F.C. Aragão, Nelson J.L. Aragão, Maria de L. Raposo, Mauro R. Elkhoury, Akemi Suzuky, Ivani B. Ferreira, Luíza Teresinha de Sousa, Cláudio S. Pannuti

**Affiliations:** * Federal University of Maranhão, São Luís, Brazil;; † Municipal Secretary of Health, Anajatuba, Brazil;; ‡ Maranhão State Quality of Life Management, São Luís, Brazil;; § Ministry of Health, Brasilia, Brazil;; ¶ Instituto Adolfo Lutz, São Paulo, Brazil;; # University of São Paulo Medical School, São Paulo, Brazil

**Keywords:** hantaviruses, hantavirus disease, hantavirus pulmonary syndrome, Anajatuba, Brazil, dispatch

## Abstract

In 2000, the first outbreak of hantavirus pulmonary syndrome was recognized in the Brazilian Amazon (Maranhão State). An epidemiologic study identified a 13.3% prevalence of hantavirus-specific immunoglobulin G. The analysis of risk factors suggests that persons are occupationally exposed to infected rodents in the crop fields.

Hantavirus pulmonary syndrome (HPS), caused by a hantavirus later identified as Sin Nombre virus, was identified for the first time in May 1993 in the southwestern United States (1). The natural reservoirs of members of *Hantavirus*, a genus belonging to the *Bunyaviridae* family, are wild rodents of the Rodentia order, Muridae family, and Sigmodontina subfamily. The human disease is a zoonosis and is acquired by inhaling aerosols containing urine, feces, or saliva particles from infected wild rodents (2–4). The disease has been described in North, Central, and South America (4).

In 2000, the first outbreak of HPS occurred in the Brazilian Amazon region (5), specifically in Quebra and São Jerônimo, in a rural area of Anajatuba, state of Maranhão, Brazil (Figure). These two villages combined had a population of 535 inhabitants. The climate is semihumid tropical, and the main economic activities are raising cassava, rice, and corn on large plantations and fishing.

**Figure:**
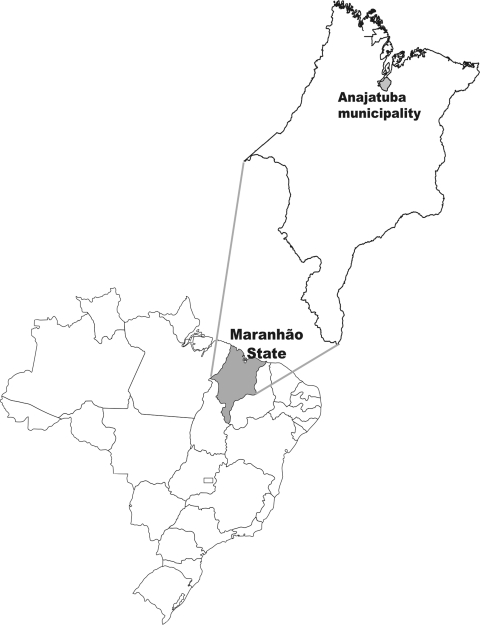
Map showing Anajatuba municipality, Maranhão State, Brazil.

## The Study

All of the inhabitants (or their legal guardians) in both towns who provided blood samples and signed the written and informed consent were included in the study. Those who did not provide blood samples were excluded (n = 137, 25.6%). No statistically significant differences were found with respect to sex and age between those studied and those excluded.

The study was conducted in two stages. First, we performed a cross-sectional analysis to determine the prevalence of hantavirus-specific immunoglobulin (Ig) G and to identify risk factors for human infection by a hantavirus. The portion of the population whose blood samples showed hantavirus antibodies were considered seropositive. In the second stage, 6 to 24 months after the first collection, we retested the portion of the population whose blood samples did not show hantavirus antibodies (seronegative cohort).

The measure of association used was the prevalence rate ratio (PRR) at the 95% confidence interval (CI). The Wald test was also used, and statistical significance was set at the 0.05 level. Those variables with p < 0.20 in the unadjusted analysis were included in the adjusted analysis. The variables with p < 0.10 were maintained in the final model after stepwise backward elimination was performed. Because prevalence of infection was >10%, the results were adjusted for confounding factors by using the Poisson regression model. Standard errors were adjusted according to the robust method, and the cluster effect was taken into account.

We used a hierarchical modeling strategy, in which the variables were divided into three blocks: block 1, socioeconomic variables (education, marital status, occupation [farm worker or housewife]); block 2, behavioral variables (storing grains inside the home, fishing, using dead rats for fishing bait, bathing in rivers, drinking water from streams or rivers, sweeping the home, seeing rats at home or in the wild, seeing rat feces inside the house, having the ability to recognize wild rats, killing a rat either at home or in the field, being bitten by a rat); and block 3, demographic variables (sex and age). The adjusted analysis was performed in three steps. In the first step, the PRR of the socioeconomic variables (block 1) was adjusted; in the second step, the PRR of the behavioral variables (block 2) was adjusted for the statistically significant variables in the first step. Finally, in the third step, the PRR of the demographic variables (block 3) was adjusted for the statistically significant variables in the second step.

Antibodies of the IgG class were detected by enzyme-linked immunosorbent assay (ELISA), by using antigen of Sin Nombre virus (Centers for Disease Control and Prevention, Atlanta, GA). The serologic tests were performed in the Department of Viruses Transmitted by Arthropods at the Instituto Adolfo Lutz, São Paulo. The samples of human serum underwent a series of dilutions and were tested for recombinant nucleocapsid protein antigen of Sin Nombre virus and for the control recombinant antigen. One conjugate of antihuman IgG, prepared in mice and marked with peroxidase and the chromogen ABTS (2,2-azino-di [3-ethybenthiazoline sulfonate]), was used to show the reaction. Samples were considered positive when they showed an optical density higher than the value of the reactivity limit at a dilution of >1:400.

Of the 535 residents of Quebra and São Jerônimo, 398 (74.4%) participated in the study. The overall seroprevalence was 13.3% (95% CI 10.1%–17.1%).

In the unadjusted analysis, age >17 years, being illiterate, living in consensual union, working as an agricultural laborer, fishing, using dead rats as bait for fishing, house sweeping, and killing rats in the field or inside the home were all significantly associated with infection by hantavirus. Those who had seen rats in the fields, had been bitten by a rat, or could recognize wild rats also were more likely to become infected (Table 1).

**Table 1:** Unadjusted analysis of risk factors for hantavirus infection in Anajatuba, Maranhão State, Brazil, 2000

Variable	PRR (95% CI)^a^
Male vs. female	1.29 (0.77–2.17)
Age (y)	
18–40 vs. <17	
41–64 vs. <17	13.4 (5.80–30.9)
>65 vs. <17	17.2 (6.62–44.5)
Living with a companion versus living alone	3.62 (2.22–5.93)
Being illiterate	3.33 (1.97–5.62)
Being a farm worker	3.65 (1.90–7.00)
Being a housewife	1.83 (1.10–3.03)
Seeing rats in the wild	5.94 (2.11–16.7)
Being bitten by a rat	3.19 (1.82–5.59)
Being able to recognize wild rats	3.18 (1.69–6.01)
Using dead rats for fishing bait	2.87 (1.20–6.85)
Fishing	2.61 (1.22–5.57)
Sweeping the home	2.36 (1.04–5.32)
Killing a rat in the field	2.02 (1.22–3.35)
Killing a rat at home	1.99 (1.14–3.47)
Seeing rats at home	1.55 (0.76–3.17)
Bathing in streams	1.55 (0.98–2.46)
Seeing rat feces inside the home	1.28 (0.78–2.10)
Storing grains inside the home	1.08 (0.53–2.20)

^a^PRR, prevalence rate ratio; CI, confidence interval.

The Poisson regression analysis was done in three steps. In the first step (testing the significance of socioeconomic factors), illiteracy, consensual union, and agricultural work were associated with hantavirus infection. In the second step (studying the effect of behavioral variables), seeing rats in the field conferred a higher risk of infection. In the third step (assessing effects of demographic variables), age >17 years was associated with hantavirus infection (Table 2).

**Table 2:** Adjusted analysis of risk factors for hantavirus infection in Anajatuba, Maranhão State, Brazil, 2000

Variables	PRR (95% CI)^a^	p
First step^b^		
Illiterate		0.001
No	1	
Yes	2.49 (1.45–4.26)	
Farm worker		0.025
No	1	
Yes	2.44 (1.12–5.32)	
Living with a companion		0.022
Yes	1	
No	2.05 (1.10–3.80)	
Second step^c^		
Seeing rats in the field		0.013
No	1	
Yes	4.22 (1.36–13.11)	
Third step^d^		
Age group (y)		< 0.001
<17	1	
18–40	3.65 (1.34–9.94)	
41–64	9.56 (3.65–25.04)	
>65	13.43 (4.86–37.10)	

^a^PRR, prevalence rate ratio; CI, confidence interval. ^b^Adjusted PRR of socioeconomic variables (block 1). ^c^PRR of behavioral variables (block 2), adjusted for statistically significant variables in the first step. ^d^PRR of demographic variables (block 3), adjusted for statistically significant variables in the second step.

In the second stage of the study, a cohort of 292 seronegative persons was tested initially by hantavirus-specific IgG 6 months after the initial collection, with one seroconversion. Of the 291 persons who remained seronegative, 234 were retested for antibodies 24 months after the initial collection; 4 seroconverted. The survival table estimated a probability of seroconversion in 24 months of 1.7% (95% CI 0.5%–4.3%). Among those who seroconverted, two reported fever during the follow-up period.

## Conclusions

The seroprevalence of hantavirus antibodies varies considerably according to the species of hantavirus and the rodents involved. A low prevalence of 1.7% for Sin Nombre virus antibodies was described in 1993 in the southwestern United States (6). In Central and South Argentina, where the genotypes Lechiguanas, Hu39694, and Andes are the most important, seroprevalence was also low, varying from 0.1% to 1.5% (7). A high prevalence, such as that observed in the area of Anajatuba, has also been described in other regions of the Americas. In the northern region of Argentina, where Orán is the most important genotype, seroprevalence is >20%. In Chile, where Andes virus predominates, a seroprevalence as high as 7.5% has been observed (8). In Paraguay, where Laguna Negra virus is the most important, the analysis of a nonrandom sample found a seroprevalence of 12.8%, while in indigenous communities a prevalence of up to 57% has been found (9). In Brazil, a serologic study in three cities in São Paulo, where Juquitiba virus was associated with HPS, detected a seroprevalence of 0.4% to 4.5% (10).

A case-control study in the southwestern United States, to examine risk factors associated with HPS, showed no association between sex, age, and HPS (11). HPS patients were more likely to have observed rodents near the home, to have stored food in the home, and to have cleaned food storage areas. In our study, age >17 years as well as being illiterate and living in a consensual union were associated with infection by hantavirus in the adjusted analysis. However, storing food in the home was not associated with a greater seroprevalence.

The risk for exposure at home versus risk for occupational exposure must be clarified. In Anajatuba, the only behavioral variable that was independently associated with hantavirus infection was seeing rats in the crop fields, adding evidence to the theory that this disease could be linked to occupational exposure.

Hantavirus transmission to humans through wild rodent bites has been reported in cases of hemorrhagic fever with renal syndrome (12). Among those who reported rat bites, seroprevalence was 38.1%, p < 0.001 in the unadjusted analysis. However, in the adjusted analysis, this variable had a borderline association with seroprevalence.

Follow-up results from a seronegative cohort demonstrated that none of the persons that seroconverted met the criteria that would define a case of HPS, indicating that mild or asymptomatic clinical forms of the disease developed with greater frequency in those who became infected than did the classic form of HPS.

The results we observed must interpreted with caution because of the small population studied and the possibility of colinearity, since many of the variables correspond to activities with a similar potential for rodent exposure. Risk factors may vary according to the virus involved. The possibility of having a mixed group of case-patients exists because the antigen detects different hantaviruses.
